# Long-Term Health-Related Quality of Life (QOL) after Paediatric Spinal Deformity Surgery and Comparison with the General Population

**DOI:** 10.3390/jcm12227142

**Published:** 2023-11-17

**Authors:** Athanasios I. Tsirikos, Silvia García-Martínez

**Affiliations:** Scottish National Spine Deformity Centre, Royal Hospital for Children and Young People, Edinburgh EH16 4TJ, UK; silvia.garcia@nhslothian.scot.nhs.uk

**Keywords:** scoliosis, adolescent idiopathic scoliosis, congenital scoliosis, syndromic scoliosis, Scheuermann kyphosis, spondylolisthesis, outcomes, SRS-22 questionnaire, EQ-5D questionnaire, VAS

## Abstract

QOL questionnaires assess patients’ perception on surgical outcomes. We reviewed 1354 patients with spinal deformity. Four hundred and twenty-eight patients had >10 years of follow-up. The SRS-22r questionnaire was completed before surgery, at 6/12/24 months, 5–10 years and >10 years postoperatively. Patients with >10 years of follow-up completed the EQ-5D VAS/index and the VAS for back/leg pain. We used QOL data reporting in the general population of 20–29 and 30–39 years of age to compare against our patient cohort. Among the patients, 993 had AIS, 80 congenital scoliosis, 102 syndromic or secondary scoliosis, 105 Scheuermann kyphosis and 40 low-grade and 34 high-grade spondylolisthesis. SRS-22r total and domain scores improved from preoperative to follow-up in all diagnosis categories. At >10 years after surgery, patients with congenital scoliosis and Scheuermann kyphosis had better SRS-22r total/domain and EQ-5D (index/VAS) scores along with lower VAS back/leg pain scores compared to the other groups. Patients with congenital scoliosis and Scheuermann kyphosis had comparable SRS-22r total/domain, EQ-5D (index/VAS) and VAS back/leg pain scores to the general population in the 20–29 year category and better scores than the 30–39 year group. Patients with AIS, syndromic/secondary scoliosis and low/high-grade spondylolisthesis had reduced SRS-22r total/domain and EQ-5D (index/VAS) scores and higher VAS back/leg pain scores compared to the 20–29 year group but comparable scores to the 30–39 year group. Patients with spinal deformity reported improved QOL and high satisfaction after surgery which was maintained at >10 years of follow-up. Patients with congenital scoliosis and Scheuermann kyphosis had better QOL outcomes (comparable to the general population of similar age) as opposed to other types of scoliosis or lumbosacral spondylolisthesis.

## 1. Introduction

The development of spinal deformity in children and adolescents can affect their quality of life as it may produce pain or have an impact on their function, self-image and mental health [1]. Spinal deformity surgery has progressed considerably in the last few decades due to the development of new techniques along with the advent of third-generation instrumentation. This evolution has allowed better curve correction with the expectation that it will correspond to improved patient-reported outcomes. The success of surgery has traditionally been assessed using clinical and radiographic parameters. The percentage of curve correction has been the benchmark to define a satisfactory surgical result. However, it is recognised that radiological curve improvement does not necessarily correlate with the patients’ perception of clinical outcomes [2,3].

Validated questionnaires such as the EuroQol 5-dimensions (EQ-5D), the Scoliosis Research Society 22r (SRS-22r) instrument and the Visual Analogue Scale (VAS) for back and leg pain were developed as patient-oriented outcome measures. These are designed to capture the impact of health status including the disease and its treatment in three fundamental domains that identify subjective multi-dimensional quality of life (QOL): physical, psychological and social functioning [4]. 

Long-term health-related QOL results have been reported in patients with adolescent idiopathic scoliosis (AIS) treated with Harrington rods or all-hook or hybrid hook/screw constructs and in those patients with spondylolisthesis treated with un-instrumented in situ fusion [5,6,7,8,9,10,11]. To the authors’ knowledge, there is no previous study presenting the long-term functional outcomes after surgical treatment of patients with AIS using all-pedicle-screw instrumentation. In addition, there is no previous report on the long-term QOL in patients treated for congenital, syndromic and secondary scoliosis, Scheuermann kyphosis or after instrumented stabilisation of lumbosacral spondylolisthesis. 

The purpose of this study was: (a) to evaluate the QOL outcomes of paediatric patients who underwent spinal deformity surgery across a range of underlying conditions and had longitudinal follow-up from preoperative to 6, 12, 24 months, 5–10 years and >10 years postoperative; (b) to compare the long-term (>10 years) QOL results between the different types of spinal deformity; (c) to compare the long-term (>10 years) QOL patient-reported outcomes to normative data recording health-related QOL of the general population. 

## 2. Materials and Methods

We performed a retrospective analysis of prospectively collected data in all patients with different types of scoliosis, Scheuermann kyphosis and spondylolisthesis who underwent surgical treatment from 2003–2021 under the care of the senior author and had complete health-related QOL assessment data. The primary outcome measure in this study was the patients’ perception of clinical outcomes which was reviewed using the SRS-22r questionnaire before surgery, at 6, 12, 24 months, 5–10 years and >10 years from the procedure. All patients had a minimum 2-year postoperative follow-up. 

In addition to the SRS-22r questionnaire, patients who had follow-up >10 years postsurgery also completed the EQ-5D (VAS and index) questionnaire and the VAS for back and leg pain. The patients’ demographic, clinical and QOL data were inputted in the British Spine Registry. The data were analysed anonymously by the Data Coordinator for our service. Informed consent was obtained from all patients involved in this study. We used normative data reporting the QOL of the general population for the age groups 20–29 years and 30–39 years as a baseline for comparison against the data that were recorded from the patients with spinal conditions included in the current study [12]. As this study provides evaluation of our service and does not have any impact on patient treatment, approval by the institutional review board was not required.

The patients with scoliosis were divided into those having AIS, congenital or syndromic scoliosis and scoliosis secondary to intraspinal anomalies (Chiari I malformation and/or syringomyelia), congenital cardiac disease/open cardiac surgery or an intraspinal tumour (Table 1).

Patients with lumbosacral spondylolisthesis were divided into low and high grade. In the high-grade spondylolisthesis group, 6 patients were operated in our service between 1995 and 2002 and as they had complete QOL assessment data pre- and postoperatively they were included in this study.

### 2.1. Questionnaires

The *SRS-22r* instrument has been used to measure QOL before and after treatment and this has been validated in patients with AIS [13]. The same questionnaire has been used to quantity patients’ perception of clinical outcomes in other types of spinal deformity in the absence of disease-specific QOL outcome measures [14]. The SRS-22r comprises 22 questions, which are divided into 5 domains assessing function, pain, self-image, mental health and patient satisfaction. Each question has 5 answers with the scoring ranging from 0 (worst score) to 5 (best score). An index number is calculated for each individual domain and an accumulative figure is generated for all domains combined to represent the total score. The satisfaction domain is only completed by patients who have received spinal treatment. Therefore, in this study we report the SRS-22r sub-total score that excludes the satisfaction domain, similar to previous studies [15]. 

The *EQ-5D* is a general non-disease-specific assessment questionnaire where individuals record their health across 5 categories in a descriptive system (index) reporting on mobility, self-care, usual activities, pain/discomfort and anxiety/depression [16,17]. The patients included in this study rated their health in each category and their answers generated an index figure that summarised their reported general health. The UK standard tariff was used as reference with the EQ-5D index score ranging between −0.59 (worst) and 1.0 (best) [18]. In addition, the EQ-5D VAS allowed the patients to score their health on a visual vertical scale with 100 being the best and 0 the worst result. 

The *VAS* is a rating system that allows patients to score the intensity of their back and leg pain. A continuous 10 cm line between 0 (no pain) on the left and 10 (worst pain) on the right is used for this calculation [19].

### 2.2. Statistical Analysis

Data were analysed using IBM SPSS v. 27.0 (Armonk, NY, USA). An independent samples *t*-test compared continuous parametric outcome questionnaire data between groups. Two-tailed *p*-values were reported, with significance at *p* < 0.05. 

## 3. Results

One thousand three hundred and fifty-four patients with scoliosis, Scheuermann kyphosis or spondylolilsthesis fulfilled the inclusion criteria as they had complete QOL data preoperatively and at a minimum 2 years postsurgery to participate in this study. Among them, 993 had AIS, 80 patients had congenital scoliosis, 102 patients had syndromic or secondary scoliosis, 105 patients had Scheuermann kyphosis and 40 patients had low-grade and 34 patients had high-grade spondylolisthesis. Four hundred and twenty-eight patients had >10 years of follow-up after surgery (mean: 15 years, range: 11–28 years).

A. Patients with AIS

Eight hundred and twenty-seven patients were female and one hundred and sixty-six male. Mean age at surgery was 15.2 years (range: 11.3–17.4). Mean age at follow-up was 29.5 years (range: 23.5–37 years). Mean follow-up after surgery was 14.5 years (range: 11–20 years). Nine hundred and seventy-four patients had a posterior spinal fusion, seven patients an anterior spinal fusion and twelve patients a combined one-stage antero-posterior fusion. All-pedicle-screw instrumentation was used in patients who had posterior or antero-posterior fusion. Two hundred and twenty patients had >10 years of follow-up. 

The SRS-22r total and individual scores improved from preoperative to 2-year postoperative follow-up (*p* < 0.001, Table 2). There was a small reduction in the SRS-22r scores from the 2-year to the >10-year review with mean satisfaction at latest follow-up of 4.68 (range: 3–5). The EQ-5D index and VAS scores were 0.83 and 83.64, respectively; the VAS scores for back and leg pain were 1.62 and 0.9 at >10-year follow-up.

B. Patients with congenital scoliosis

Forty-one patients were male and thirty-nine female. Mean age at surgery was 14.6 years (range: 11.1–19.5). Mean age at follow-up was 28.3 years (range: 20–35 years). Mean follow-up after surgery was 15.6 years (range: 12–21 years). Eighty-five patients had a posterior spinal fusion and five patients a combined one-stage antero-posterior fusion. All-pedicle-screw or hybrid pedicle hook/screw instrumentation was used in all patients. Forty-five patients had >10 years of follow-up. 

The SRS-22r total and individual scores improved sequentially from preoperative to >10-year postoperative follow-up (*p* < 0.001, Table 3) with mean satisfaction at latest follow-up of 4.93 (range: 4.5–5). The EQ-5D index and VAS scores were 0.91 and 87.7, respectively; the VAS scores for back and leg pain were 0.75 and 0.3 at >10-year follow-up.

C. Patients with syndromic/secondary scoliosis

Fifty-five patients were female and forty-seven male. Mean age at surgery was 15 years (range: 12–17.1). Mean age at follow-up was 30.2 years (range: 28–33 years). Mean follow-up after surgery was 14.5 years (range: 12–19.3 years). Ninety patients had a posterior spinal fusion using all-pedicle-screw or hybrid pedicle hook/screw instrumentation and one patient had an anterior spinal fusion. Thirty-six patients had >10 years of follow-up. 

The SRS-22r total and individual scores improved from preoperative to 2-year postoperative follow-up (*p* < 0.001, Table 4). There was a small reduction in the SRS-22r scores from the 2-year to the >10-year review with mean satisfaction at latest follow-up of 4.64 (range: 4–5). The EQ-5D index and VAS scores were 0.84 and 83.2, respectively; the VAS scores for back and leg pain were 1.69 and 1.1 at >10-year follow-up.

D. Patients with Scheuermann kyphosis

Sixty-one patients were male and forty-four female. Mean age at surgery was 16.5 years (range: 12.2–17.5). Mean age at follow-up was 30.1 years (range: 25–34 years). Mean follow-up after surgery was 15 years (range: 12–18 years). One hundred and three patients had a posterior spinal fusion and two patients had a combined one-stage antero-posterior spinal fusion. Hybrid pedicle hook/screw instrumentation was used in all patients. Sixty-nine patients had >10 years of follow-up. 

The SRS-22r total and individual scores improved from preoperative to 2-year postoperative follow-up (*p* < 0.001, Table 5) and then remained stable to the >10-year review with mean satisfaction at latest follow-up of 4.85 (range: 3.5–5). The EQ-5D index and VAS scores were 0.89 and 87.69, respectively; the VAS scores for back and leg pain were 0.61 and 0.57 at >10-year follow-up.

E. Patients with low-grade isthmic spondylolisthesis

Twenty-six patients were female and fourteen male. Mean age at surgery was 14.6 years (range: 10.3–17.3). Mean age at follow-up was 27.1 years (range: 21.1–33 years). Mean follow-up after surgery was 14.4 years (range: 11–17.3 years). All patients underwent a posterolateral lumbosacral fusion with autologous iliac crest bone graft [20]. Thirty patients had >10 years of follow-up.

The SRS-22r total and individual scores improved from preoperative to 2-year postoperative follow-up (*p* < 0.001, Table 6) and then remained stable to the >10-year review with mean satisfaction at latest follow-up of 4.69 (range: 3–5). The EQ-5D index and VAS scores were 0.83 and 83.4, respectively; the VAS scores for back and leg pain were 1.74 and 1.75 at >10-year follow-up.

F. Patients with high-grade dysplastic spondylolisthesis

Twenty-five patients were male and nine female. Mean age at surgery was 13.8 years (range: 10.3–17.8). Mean age at latest follow-up was 37 years (range: 25–55 years). Mean follow-up after surgery was 21 years (range: 12–28 years). All patients underwent a posterior lumbosacral fusion using the transfixation S1 to L5 screw technique and autologous iliac crest bone graft [21]. Twenty-eight patients had >10 years of follow-up.

The SRS-22r total and individual scores improved from preoperative to 2-year postoperative follow-up (*p* < 0.001, Table 7). There was a small reduction in the SRS-22r scores from the 2-year to the >10-year review with mean satisfaction at latest follow-up of 4.82 (range: 4.5–5). The EQ-5D index and VAS scores were 0.81 and 82.6, respectively; the VAS scores for back and leg pain were 1.82 and 1.8 at >10-year follow-up.

### 3.1. Comparison of QOL among Different Spinal Conditions

At >10-year follow-up after surgery, patients with congenital scoliosis had better SRS-22r total and domain scores compared to the other groups followed by patients with Scheuermann kyphosis (*p* < 0.01, Table 8). Patients with AIS, syndromic/secondary scoliosis and high- and low-grade spondylolisthesis had no difference in their total or individual domain scores at latest follow-up (*p* > 0.05).

Similarly, patients with congenital scoliosis and Scheuermann kyphosis reported better EQ-5D (index/VAS), as well as lower VAS for back/leg pain scores compared to the other four groups (*p* < 0.01). There was no difference in these scores among patients with AIS, syndromic/secondary scoliosis and high- and low-grade spondylolisthesis at latest follow-up (*p* > 0.05).

### 3.2. Comparison of QOL between Patients with Spinal Conditions and the General Population

**Figure 1:**
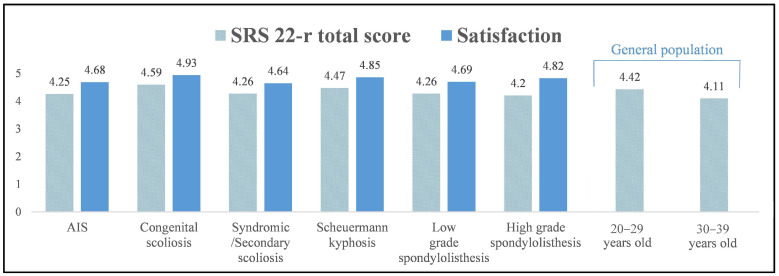
Mean SRS-22r total scores and satisfaction at >10-year follow-up in the groups of patients with different types of spinal deformity and comparison to the SRS-22r total scores of the general population in the 20–29- and 30–39-year-old groups.

Patients with congenital scoliosis and Scheuermann kyphosis who were >10 years after surgery had comparable SRS-22r total and domain scores to the general population in the 20–29 year category (*p* > 0.05) and better scores than the 30–39 year group (*p* < 0.001) (Table 9, Figure 1). In contrast, patients with AIS, syndromic/secondary scoliosis and low- and high-grade spondylolisthesis had reduced SRS-22r total and domain scores when compared to the 20–29 year group of the general population (*p* < 0.01) but comparable scores to the 30–39 year group (*p* > 0.05).

**Figure 2:**
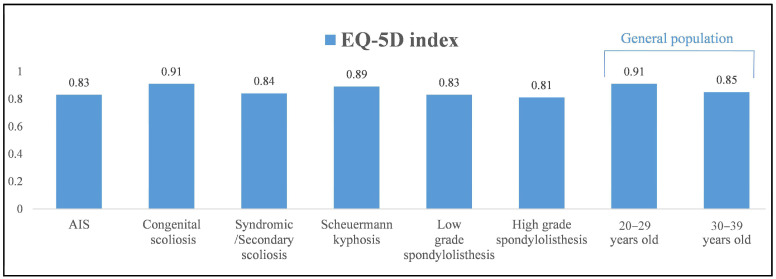
Mean EQ-5D index scores at >10-year follow-up in the groups of patients with different types of spinal deformity and comparison to those of the general population in the 20–29- and 30–39-year-old groups.

**Figure 3:**
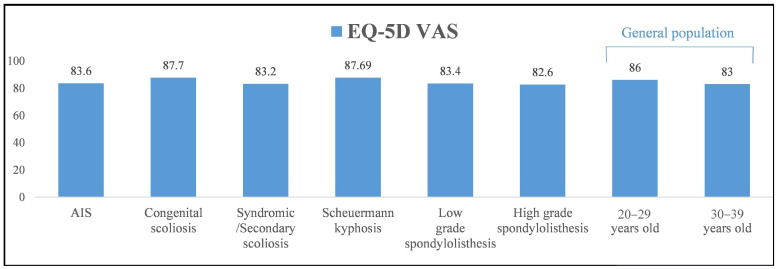
Mean EQ-5D VAS scores at >10-year follow-up in the groups of patients with different types of spinal deformity and comparison to those of the general population in the 20–29- and 30–39-year-old groups.

**Figure 4:**
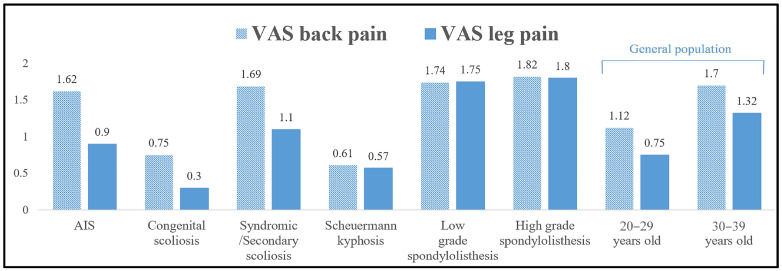
Mean VAS for back and leg pain scores at >10-year follow-up in the groups of patients with different types of spinal deformity and comparison to those of the general population in the 20–29- and 30–39-year-old groups.

Patients with congenital scoliosis and Scheuermann kyphosis had comparable EQ-5D (index/VAS) scores as well as VAS for back/leg pain scores to the 20–29 year group when using the normative general population data (*p* > 0.05) and better scores than the 30–39 year category (*p* < 0.001, Table 10, Figure 2, Figure 3 and Figure 4). In contrast, patients with AIS, syndromic/secondary scoliosis and low- and high-grade spondylolisthesis reported lower EQ-5D (index/VAS) and higher VAS (back/leg pain) scores than the 20–29 year group (*p* < 0.01) and comparable scores to the 30–39 year group from the general population (*p* > 0.05).

## 4. Discussion

Scoliosis left untreated at skeletal maturity can deteriorate into adult life, affecting the patients’ QOL compared to the general population and causing cardio-respiratory impairment [22]. Weinstein et al. [23] reported on the natural history of idiopathic scoliosis in patients who did not receive treatment. Of patients with thoracic curves >80°, 22% had shortness of breath that impacted on activities of daily living; 61% of patients complained of chronic back pain with 68% of them having low or moderate intensity. Farshad et al. [24] compared the outcomes of surgically versus non-surgically treated patients with moderate AIS after a minimum of 29 years of follow-up and noted similar subjective results regarding function and disability with a smaller curve size in the operated group.

Scoliosis correction requires long fusions that can compromise spinal flexibility and increase the risk of lumbar degeneration as a consequence of undue stresses placed on the caudal un-instrumented levels. The incidence of low back pain at medium-term follow-up after spinal fusion using Cotrel–Dubousset instrumentation has been reported to range between 35% and 45% [25,26]. Previous series with longer postoperative follow-up of >20 years documented occasional or frequent back pain in 15% and 25% of patients with no major dysfunction and no difference in general health-related QOL compared to controls unaffected by scoliosis [6,8,27]. Despite the fact that the initial studies recorded outcomes using older correction techniques and first-generation instrumentation, health-related QOL was maintained with good reported self-image and mental health [5,8]. Kibsgard et al. [28] found that while scoliosis surgery patients had slightly lower physical health scores compared to their peers who did not have scoliosis, their mental health scores were actually higher. 

The evolution of scoliosis surgery has focused on the use of extensive spinal releases and modern instrumentation techniques that have achieved better deformity correction while limiting the number of fusion levels with the aim to reduce the risk of distal degeneration and optimise patient function. Green et al. [29] and Darnis et al. [9] reported good functional scores and QOL not dissimilar to the general population with little loss of scoliosis correction in patients with AIS treated with hybrid instrumentation and followed for 10 and 20 years postsurgery, respectively. The degenerative process below the levels of the fusion in these two series remained relatively rare and was found remote to the lowest instrumented vertebra in the lumbosacral disc without affecting the functional outcome [9,29].

The present study is the first to report long-term health-related QOL in patients with scoliosis treated with all-pedicle-screw instrumentation. Patients in the AIS and non-AIS groups had better total and domain SRS-22r scores compared to preoperative and these were maintained at >10 years after surgery. Patients with Scheuermann kyphosis treated with posterior closing wedge osteotomies and hybrid instrumentation, as well as those with low- and high-grade lumbosacral spondylolisthesis, also demonstrated significant improvement in their SRS-22r scores between preoperative and long-term follow-up. The improved SRS-22r total and individual domain scores were associated in all groups with high patient satisfaction. 

At latest follow-up >10 years after surgery, patients with congenital scoliosis and Scheuermann kyphosis had better SRS-22r (total/domain scores), EQ-5D (index/VAS scores), as well as VAS (back/leg pain) scores compared to the other groups. There is no previous study that can validate these results in patients with severe congenital scoliosis or Scheuermann kyphosis. However, we only treated patients with severe and progressive Scheuermann kyphosis >80° who had persistent back pain and genuine complaints regarding their cosmetic appearance. It is likely that this is the reason why their long-term health-related outcomes and treatment satisfaction were so positive. Equally, patients with congenital scoliosis often have associated medical co-morbidities requiring additional treatments at a young age and commonly throughout their lives. It is possible that these patients have a higher appreciation of a positive surgical outcome following scoliosis treatment than the AIS group in the context of their complex medical history. 

In a previous comparative series, Helenius et al. [11] recorded better long-term health-related QOL outcomes including self-image and function in patients with AIS compared to patients who had surgery for spondylolisthesis. In contrast, in the current study we noted no difference in reported QOL at >10 years from surgery using the SRS-22r, EQ-5D and VAS questionnaires among patients with AIS, syndromic/secondary scoliosis and low- or high-grade spondylolisthesis. 

In addition, at the last assessment >10 years postsurgery patients with congenital scoliosis and Scheuermann kyphosis with mean age 28.3 and 30.1 years, respectively, had SRS-22r (total/domain scores), EQ-5D (index/VAS scores) and VAS (back/leg pain) scores that were as good as the general population in the 20–29 year group and better than those of the 30–39 year group. This finding indicates that surgically treated patients with congenital scoliosis or Scheuermann kyphosis have similar function and health-related QOL compared to the general population that has no spinal deformity. We used previously published normative values that are country-specific for the SRS-22r, EQ-5D (index/VAS) and VAS for back/leg pain questionnaires in the adult general population of the United Kingdom as these provide the ability for direct comparison to our patient cohorts [12]. 

Patients with AIS, syndromic/secondary scoliosis and low- or high-grade spondylolisthesis with mean age at >10 years after surgery of 29.5, 30.2, 27.1 and 37 years, respectively, had SRS-22r (total/domain scores), EQ-5D (index/VAS scores) and VAS (back/leg pain) scores comparable to the 30–39 year group but worse than the 20–29 year group of the general population. This finding suggests that spinal surgery did not affect patient-perceived health-related outcomes as patients of all underlying diagnoses included in this study had good quality of life that was not different to their unaffected peers. 

A limitation of this study is that, despite the long available postoperative follow-up, the patients included in the different groups were still young at last review. Therefore, they could develop functional problems or back pain related to their underlying spinal deformity or the surgery that they underwent in later life. There is an argument that this evolution of back-related symptoms over time occurs in the general population as part of the normal ageing process leading to progressive spinal degeneration. Nevertheless, we plan to continue monitoring this group of patients as they progress into their middle and senior adult life to understand any impact that their spinal history may have on their long-term health. 

In conclusion, in this study we reported good long-term QOL that significantly improved in all functional categories from preoperative to a mean of 15 years after surgery. This was associated with normal function, good self-image, less pain and high patient satisfaction in all individual spinal deformity groups. The majority of patients was satisfied with the outcome of their treatment and went on to have productive lives with no limitations, similar to the general population. Patients with congenital scoliosis and Scheuermann kyphosis had better health-related QOL compared to those patients with AIS, other types of scoliosis or spondylolisthesis. These data can be used as a reference in order to manage patients’ expectations while informing their decision regarding surgical treatment and its anticipated long-term outcomes. It can also assist with public health strategies in the management of spinal deformity conditions with the aim to limit their impact on long-term morbidity and the development of chronic back pain. 

## Figures and Tables

**Table 1:** Underlying diagnosis in the group of patients with a secondary or syndromic scoliosis who underwent surgical correction.

Diagnosis	Secondary Scoliosis	Syndromic Scoliosis
Congenital cardiac disease	21	-
Syringomyelia	18	-
Chiari I malformation/syringomyelia	26	-
Chiari I malformation	7	-
Congenital cardiac disease, Chiari I malformation/syringomyelia	1	-
Spinal cord tethering	1	-
Marfan syndrome	-	4
Neurofibromatosis type I	-	4
Di George syndrome	-	2
Osteogenesis imperfecta (OI)	-	2
Klippel–Trenaunay–Weber syndrome	-	2
Turner syndrome	-	2
Undiagnosed syndrome	-	2
Asperger’s syndrome	-	1
Ehlers–Danlos syndrome	-	1
Chromosome abnormality	-	2
Coffin–Siris syndrome	-	1
Dandy–Walker syndrome	-	1
Opitz G syndrome	-	1
Intraspinal tumour	2	-
Pilocytic astrocytoma	1	-

**Table 2:** SRS-22r questionnaire outcomes in the AIS group.

AIS Group	Function	Pain	Self-Image	Mental Health	Satisfaction	Total Score
Preoperative (993 patients)	4.04 (range: 1.8–5)	3.86 (range: 1.4–5)	3.16 (range: 2–5)	3.84 (range: 1.4–5)	-	3.7 (range: 1.95–5)
6 months postoperative (993 patients)	3.81 (range: 1.8–5)	4.24 (range: 1.4–5)	4.25 (range: 2–5)	4.1 (range: 1.4–5)	4.77 (range: 1.5–5)	4.15 (range: 1.09–5)
12 months postoperative (993 patients)	4.29 (range: 2.6–5)	4.42 (range: 1.4–5)	4.28 (range: 2–5)	4.23 (range: 1–5)	4.81 (range: 2.5–5)	4.35 (range: 2.41–5)
24 months postoperative (993 patients)	4.43 (range: 2.4–5)	4.51 (range: 2.2–5)	4.32 (range: 1.2–5)	4.2 (range: 1.4–5)	4.78 (range: 2–5)	4.4 (range: 1.95–5)
5–10 years postoperative (560 patients)	4.39 (range: 1.2–5)	4.45 (range: 1.2–5)	4.32 (range: 1–5)	4.01 (range: 1–5)	4.77 (range: 1–5)	4.44 (range: 1.55–4.95)
>10 years postoperative (220 patients)	4.29 (range: 2–5)	4.35 (range: 1.2–5)	4.21 (range: 2–5)	3.99 (range: 1.8–5)	4.68 (range: 3–5)	4.25 (range: 2.18–5)

**Table 3:** SRS-22r questionnaire outcomes in the congenital scoliosis group.

Congenital Scoliosis Group	Function	Pain	Self-Image	Mental Health	Satisfaction	Total Score
Preoperative (80 patients)	3.98 (range: 2.4–4.8)	3.94 (range: 1.8–5)	3.22 (range: 1.6–4.6)	3.98 (range: 1.2–5)	-	3.77 (range: 1.75–4.6)
6 months postoperative (80 patients)	3.67 (range: 1.6–4.8)	4.23 (range: 2.8–5)	3.91 (range: 2.6–4.8)	4.28 (range: 2.2–5)	4.77 (range: 3–5)	4.09 (range: 2.55–4.68)
12 months postoperative (80 patients)	3.98 (range: 2.4–4.8)	4.37 (range: 2.8–5)	4.08 (range: 2–5)	4.11 (range: 2–5)	4.86 (range: 3–5)	4.2 (range: 2.36–4.77)
24 months postoperative (80 patients)	4.39 (range: 3.4–4.8)	4.46 (range: 2.6–5)	4.08 (range: 2.4–5)	4.16 (range: 1.8–5)	4.64 (range: 2–5)	4.31 (range: 3–5)
5–10 years postoperative (65 patients)	4.39 (range: 3.8–4.8)	4.68 (range: 3.4–5)	4.24 (range: 2.4–5)	4.4 (range: 2.6–5)	4.75 (range: 2.5–5)	4.46 (range: 3.36–4.95)
>10 years postoperative (45 patients)	4.49 (range: 4–5)	4.63 (range: 3.8–5)	4.62 (range: 3.75–5)	4.49 (range: 3–5)	4.93 (range: 4.5–5)	4.59 (range: 4.18–4.91)

**Table 4:** SRS-22r questionnaire outcomes in the syndromic and secondary scoliosis groups.

Syndromic and Secondary Scoliosis Group	Function	Pain	Self-Image	Mental Health	Satisfaction	Total Score
Preoperative (102 patients)	3.97 (range: 3–4.8)	3.79 (range: 1.8–5)	2.99 (range: 1–5)	3.61 (range: 1–5)	-	3.58 (range: 2.09–4.6)
6 months postoperative (102 patients)	3.64 (range: 2–5)	4.22 (range: 2.2–5)	4.11 (range: 3–5)	3.97 (range: 2.6–5)	4.78 (range: 4–5)	4.06 (range: 3.27–4.91)
12 months postoperative (102 patients)	4.22 (range: 2.97–5)	4.5 (range: 3–5)	4.33 (range: 1.6–5)	4.26 (range: 1.6–5)	4.81 (range: 3.5–5)	4.37 (range: 2.91–5)
24 months postoperative (102 patients)	4.47 (range: 3.2–5)	4.67 (range: 3.6–5)	4.41 (range: 2.8–5)	4.34 (range: 1.8–5)	4.85 (range: 3.5–5)	4.51 (range: 3.49–4.95)
5–10 years postoperative (72 patients)	4.34 (range: 3–5)	4.53 (range: 3.4–5)	4.44 (range: 3.4–5)	4.32 (range: 2.8–5)	4.8 (range: 2.5–5)	4.44 (range: 3.73–4.95)
>10 years postoperative (36 patients)	4.17 (range: 2.6–4.8)	4.11 (range: 3.2–5)	4.31 (range: 3.6–5)	4.29 (range: 3.4–5)	4.64 (range: 4–5)	4.26 (range: 3.36–4.77)

**Table 5:** SRS-22r questionnaire outcomes in the Scheuermann kyphosis group.

Scheuermann Kyphosis Group	Function	Pain	Self-Image	Mental Health	Satisfaction	Total Score
Preoperative (105 patients)	3.81 (range: 1.75–5)	3.5 (range: 1.2–5)	2.82 (range: 1–5)	3.54 (range: 1.4–5)	-	3.44 (range: 1.88–4.9)
6 months postoperative (105 patients)	3.56 (range: 1.8–5)	4.23 (range: 2.4–5)	4.24 (range: 3–5)	3.94 (range: 2.4–5)	4.84 (range: 4–5)	4.07 (range: 3.18–4.95
12 months postoperative (105 patients)	4.13 (range: 2.8–5)	4.43 (range: 2.6–5)	4.35 (range: 3–5)	4.16 (range: 2.8–5)	4.8 (range: 3–5)	4.31 (range: 3.32–5)
24 months postoperative (105 patients)	4.27 (range: 2.8–5)	4.52 (range: 2.4–5)	4.41 (range: 2.4–5)	4.2 (range: 2–5)	4.82 (range: 3–5)	4.39 (range: 2.95–5)
5–10 years postoperative (82 patients)	4.46 (range: 2.2–5)	4.56 (range: 2.6–5)	4.6 (range: 3.4–5)	4.21 (range: 2–5)	4.92 (range: 4–5)	4.5 (range: 3.09–5)
>10 years postoperative (69 patients)	4.51 (range: 3.2–5)	4.5 (range: 3–5)	4.47 (range: 1.6–5)	4.27 (range: 2.2–5)	4.85 (range: 3.5–5)	4.47 (range: 2.59–5)

**Table 6:** SRS-22r questionnaire outcomes in the low-grade lumbosacral spondylolisthesis group.

Low-Grade Spondylolisthesis Group	Function	Pain	Self-Image	Mental Health	Satisfaction	Total Score
Preoperative (40 patients)	3.30 (range: 2.2–4.6)	3 (range: 2–4.6)	3.4 (range: 2.4–4.2)	3.73 (range: 1.6–4.8)	-	3.31 (range: 2.25–4.45)
6 months postoperative (40 patients)	3.85 (range: 2.4–5)	4.04 (range: 1.8–5)	4.09 (range: 2.4–5)	3.95 (range: 3.2–5)	4.89 (range: 3.5–5)	4.06 (range: 2.55–5)
12 months postoperative (40 patients)	4.21 (range: 2.6–5)	4.03 (range: 2–5)	4.17 (range: 3.2–5)	3.83 (range: 1.4–5)	4.75 (range: 3.5–5)	4.12 (range: 2.86–5)
24 months postoperative (40 patients)	4.25 (range: 2.4–5)	4.19 (range: 1–5)	4.18 (range: 2.6–5)	4.19 (range: 2–5)	4.68 (range: 4–5)	4.24 (range: 2.35–5)
5–10 years postoperative (35 patients)	4.09 (range: 1.8–5)	4.28 (range: 2–5)	4.26 (range: 2.4–5)	4.02 (range: 2–5)	4.72 (range: 4.5–5)	4.21 (range: 2.68–4.86)
>10 years postoperative (30 patients)	4.28 (range: 2.8–5)	4.37 (range: 2–5)	4.14 (range: 3–5)	4.09 (range: 2.4–5)	4.69 (range: 4–5)	4.26 (range: 2.91–4.86)

**Table 7:** SRS-22r questionnaire outcomes in the high-grade lumbosacral spondylolisthesis group.

High-Grade Spondylolisthesis Group	Function	Pain	Self-Image	Mental Health	Satisfaction	Total Score
Preoperative (34 patients)	3.49 (range: 1.8–4.4)	3.23 (range: 1.6–4.2)	3.51 (range: 2–4.5)	4.03 (range: 2.4–5)	-	3.57 (range: 2.3–4.5)
6 months postoperative (34 patients)	4.25 (range: 3.7–4.6)	4.32 (range: 3.5–4.7)	4.18 (range: 3–4.4)	4.1 (range: 3.1–4.8)	4.6 (range: 4–5)	4.23 (range: 2.85–5)
12 months postoperative (34 patients)	4.4 (range: 4.2–5)	4.43 (range: 3.8–4.65)	4.32 (range: 3.5–4.7)	4.26 (range: 3.6–5)	4.75 (range: 4–5)	4.52 (range: 3.24–5)
24 months postoperative (34 patients)	4.64 (range: 4.33–5)	4.57 (range: 3.8–5)	4.58 (range: 3.8–5)	4.35 (range: 3.6–5)	4.94 (range: 4–5)	4.57 (range: 4.23–4.91)
5–10 years postoperative (32 patients)	4.13 (range: 2.2–5)	4.04 (range: 2.2–5)	3.91 (range: 2–5)	3.95 (range: 2.2–5)	4.36 (range: 4–5)	4.04 (range: 2.14–4.82)
>10 years postoperative (28 patients)	4.19 (range: 2.4–5)	4.23 (range: 2–5)	4.08 (range: 1.6–5)	4.05 (range: 1.6–5)	4.82 (range: 4–5)	4.2 (range: 2.27–4.91)

**Table 8:** Comparison of the SRS-22r total scores at different review points from preoperative to >10-year postoperative follow-up among the different diagnosis groups.

	AIS	Congenital Scoliosis	Syndromic and Secondary Scoliosis	Scheuermann Kyphosis	Spondylolisthesis	
					High Grade	Low Grade
	(993 patients)	(80 patients)	(102 patients)	(105 patients)	(34 patients)	(40 patients)
Preoperative	3.7 (range: 1.95–5)	3.77 (range: 1.75–4.6)	3.58 (range: 2.09–4.6)	3.44 (range: 1.88–4.9)	3.57 (range: 2.3–4.5)	3.31 (range: 2.25–4.45)
6 months postoperative	4.15 (range: 1.09–5)	4.09 (range: 2.55–4.68)	4.06 (range: 3.27–4.91)	4.07 (range: 3.18–4.95)	4.23 (range: 2.85–5)	4.06 (range: 2.55–5)
12 months postoperative	4.35 (range: 2.41–5)	4.2 (range: 2.36–4.77)	4.37 (range: 2.91–5)	4.31 (range: 3.32–5)	4.52 (range: 3.24–5)	4.12 (range: 2.86–5)
24 months postoperative	4.4 (range: 1.95–5)	4.31 (range: 3–5)	4.51 (range: 3.45–4.95)	4.39 (range: 2.95–5)	4.57 (range: 4.23–4.91)	4.24 (range: 2.35–5)
5–10 years postoperative	4.34 (range: 1.55–4.95)	4.46 (range: 3.36–4.95)	4.44 (range: 3.73–4.95)	4.5 (range: 3.09–5)	4.04 (range: 2.14–4.82)	4.21 (range: 2.68–4.86)
>10 years postoperative	4.25 (range: 2.18–5.00)	4.59 (range: 4.18–4.91)	4.26 (range: 3.36–4.77)	4.47 (range: 2.59–5)	4.2 (range: 2.27–4.91)	4.26 (range: 2.91–4.86)

**Table 9:** Comparison of the SRS-22r domain and total scores at >10-year follow-up among the different diagnosis groups and the general population [12].

	AIS	Congenital Scoliosis	Syndromic and Secondary Scoliosis	Scheuermann Kyphosis	Spondylolisthesis		General Population	
					High Grade	Low Grade	20–29 Years Old	30–39 Years Old
	(220 patients)	(45 patients)	(36 patients)	(69 patients)	(28 patients)	(30 patients)	(183 people)	(115 people)
**Mean age (years)**	29.5 (range: 23.5–37)	28.3 (range: 20–35)	30.2 (range: 28–33)	30.1 (range: 25–34)	37 (range: 25–55)	27.1 (range: 21.1–33)	25 (range: 20–29)	34 (range: 30–39)
**Function**	4.29 (range: 2–5)	4.49 (range: 4–5)	4.17 (range: 2.6–4.8)	4.51 (range: 3.2–5)	4.19 (range: 2.4–5)	4.28 (range: 2.8–5)	4.51 (range: 2–5)	4.23 (range: 2.6–5)
**Pain**	4.35 (range: 1.2–5)	4.63 (range: 3.8–5)	4.11 (range: 3.2–5)	4.50 (range: 3–5)	4.23 (range: 2–5)	4.37 (range: 2–5)	4.58 (range: 1.8–5)	4.3 (range: 1.6–5)
**Self-image**	4.21 (range: 2–5)	4.62 (range: 3.75–5)	4.31 (range: 3.6–5)	4.47 (range: 1.6–5)	4.08 (range: 1.6–5)	4.14 (range: 3–5)	4.37 (range: 1.8–5)	4.04 (range: 2.4–5)
**Mental health**	3.99 (range: 1.8–5)	4.49 (range: 3–5)	4.29 (range: 3.4–5)	4.27 (range: 2.2–5)	4.05 (range: 1.6–5)	4.09 (range: 2.4–5)	4.22 (range: 2.4–5.6)	3.87 (range: 2–5)
**Total score**	4.25 (range: 2.18–5)	4.59 (range: 4.18–4.91)	4.26 (range: 3.36–4.77)	4.47 (range: 2.59–5)	4.2 (range: 2.27–4.91)	4.26 (range: 2.91–4.86)	4.42 (range: 2.42–5)	4.11 (range: 2.47–4.89)

**Table 10:** Comparison of the EQ-5D (index/VAS) and the VAS for back and leg pain at >10-year follow-up among the different diagnosis groups and the general population [12].

	AIS	Congenital Scoliosis	Syndromic and Secondary Scoliosis	Scheuermann Kyphosis	Spondylolisthesis		General Population	
					High Grade	Low Grade	20–29 Years Old	30–39 Years Old
	(220 patients)	(45 patients)	(36 patients)	(69 patients)	(28 patients)	(30 patients)	(183 people)	(115 people)
**Mean age (years)**	29.5 (range: 23.5–37)	28.3 (range: 20–35)	30.2 (range: 28–33)	30.1 (range: 25–34)	37 (range: 25–55)	27.1 (range: 21.1–33)	25 (range: 20–29)	34 (range: 30–39)
**EQ-5D VAS**	83.64 (range: 70–100)	87.7 (range: 70–99)	83.2 (range: 30–90)	87.69 (range: 60–100)	82.6 (range: 40–95)	83.4 (range: 40–99)	86 (range: 25–100)	83 (range: 40–100)
**EQ-5D index**	0.83 (range: 0.13–1)	0.91 (range: 0.8–1)	0.84 (range: 0.18–1)	0.89 (range: 0.1–1)	0.81 (range: 0.1–1)	0.83 (range: 0.16–1)	0.91 (range: 0.06–1)	0.85 (range: 0–1)
**VAS back pain**	1.62 (range: 0–9)	0.75 (range: 0–3)	1.69 (range: 0–9.2)	0.61 (range: 0–2.7)	1.82 (range: 0–6)	1.74 (range: 0–6)	1.12 (range: 0–8)	1.7 (range: 0–8)
**VAS leg pain**	0.9 (range: 0–9)	0.3 (range: 0–3)	1.1 (range: 0–8)	0.57 (range: 0–6)	1.8 (range: 0–8)	1.75 (range: 0–6)	0.75 (range: 0–9)	1.32 (range: 0–10)

## Data Availability

The health related quality of life data presented in this study is available on the British Spine Registry (BSR).
